# The path from schizotypy to depression and aggression and the role of family stress

**DOI:** 10.1192/j.eurpsy.2020.76

**Published:** 2020-07-30

**Authors:** Preethi Premkumar, Elizabeth Kuipers, Veena Kumari

**Affiliations:** 1 Department of Psychology, School of Social Sciences, Nottingham Trent University, Nottingham, United Kingdom; 2 King’s College London, Department of Psychology, Institute of Psychiatry, Psychology and Neuroscience, London, United Kingdom; 3 NIHR Biomedical Research Centre for Mental Health, South London and Maudsley NHS Foundation Trust, London, United Kingdom; 4 Centre for Cognitive Neuroscience, College of Life and Health Sciences, Brunel University London, Uxbridge, United Kingdom

**Keywords:** Arousal, criticism, disorganization, expressed emotion, path analysis, praise

## Abstract

**Background.:**

Schizotypy is a multidimensional construct that is linked to the vulnerability for psychosis. Positive schizotypy includes having paranormal beliefs. Negative schizotypy includes social anhedonia. Disorganized schizotypy includes social anxiety and communication disorder. Schizotypy relates to depression and aggression. Family stress from high expressed emotion (EE; a rating of criticism, hostility, and emotional overinvolvement in a close relative toward a person showing signs of mental disorder) may mediate the link between schizotypy, depression and aggression. This study tested, using path analyses, the hypotheses that schizotypy predicts depression and aggression through high perceived EE as criticism and irritability (hypothesis 1) and praise and intrusiveness in a close relative (hypothesis 2).

**Methods.:**

One hundred and four healthy participants listened to and rated the self-relevance of standard criticism and standard praise that denote EE. Participants rated their level of schizotypy, depression, aggression, and perceived EE in self-report questionnaires. Two path models tested the hypotheses.

**Results.:**

Disorganized schizotypy, more than positive schizotypy, predicted the path to depression and aggression when perceived criticism and perceived EE-irritability were mediators. Disorganised schizotypy, more than negative schizotypy, predicted the path to depression and aggression when perceived praise and perceived EE-intrusiveness were mediators.

**Conclusions.:**

Greater perceived criticism and less perceived praise in family communication explain the path from disorganized schizotypy (more so than positive or negative schizotypy) to depression and aggression. These findings indicate a need to consider the thought disorder-EE link as a potential contributor to depression and aggression in people with schizophrenia.

## Introduction

Schizotypy is a multidimensional construct consisting of many personality traits that are linked to subclinical experiences of psychosis [1]. Positive schizotypy consists of perceptual aberrations, paranormal beliefs, delusional beliefs, and referential thinking (here, referential thinking implies incorrectly interpreting and assigning unusual meaning to casual external events) [2]. Negative schizotypy comprises a lack of pleasure in physical and social activities and social withdrawal. Cognitive disorganization includes social anxiety, communication disorder, and having poor cognitive control [3]. Impulsive nonconformity is a fourth dimension of schizotypy that consists of violent, self-abusive, and reckless behaviors and is thought to reflect aggression [2]. However, impulsive nonconformity is not considered to be a core schizotypal trait [4]. Instead, impulsive nonconformity is considered subsidiary to positive schizotypy [5] because impulsivity and hostility seem to derive from core schizotypal traits, such as paranoia and suspiciousness [6].

### The family context of the relationship between schizotypy and aggression

Aggression is characterized by behaving in a way that intends to inflict harm upon a victim when the victim is motivated to avoid the harm [7,8]. Aggression may be imitated [9] or arise in conflictual communication, such as when romantic partners criticize each other [10]. People with schizophrenia and schizotypy, however, often show reactive aggression, rather than proactive aggression [11–13]. Such reactive aggression could come about from patients perceiving criticism and hostility in the family [13] or high expressed emotion (EE) within the family context. EE is a rating of the level of criticism, hostility, emotional overinvolvement, warmth, and/or positive comments from a family member toward the patient [14]. Patients with schizophrenia make three times as many criticisms and display more hostility when they interact with a high EE parent [15]. Individuals with high schizotypy also encounter more EE-rated hostility than individuals with low schizotypy [16]. Individuals with high schizotypy also react aggressively to peer victimization [11].

### The pathway from schizotypy to depression and aggression through perceived EE

High positive schizotypal traits are linked to negative metacognitive beliefs in depression [17]. Negative metacognitive beliefs include being self-critical [18,19]. Depression may be linked to disorganized schizotypy more strongly than to positive or negative schizotypy because disorganized schizotypy is reported to be a better predictor of negative affect than positive or negative schizotypy [20]. Disorganized schizotypy comprises “nervousness due to confusedness” [21], whereas positive schizotypal experiences do not necessarily encompass negative affect from neuroticism/anxiety [22]. The negative affect in positive schizotypy, if present, is associated with disorganized schizotypy [20]. Besides, anxiety in disorganized schizotypy is accompanied by low mood [23] which could explain the link between disorganised schizotypy and depression. Depression also relates to aggression [24], with reports of low-level depression coexisting with aggression [25]. Disorganization also appears to be linked to aggression, since disorganization is greater among patients with schizophrenia who are aggressive than nonaggressive [26].

Social anxiety from being sensitive to reward and punishment from people may mediate the relationship of schizotypy to depression and aggression. Criticism and praise are types of social punishment and social reward, respectively. Self-criticism is the tendency to set unrealistically high self-standards and adopt a punitive stance toward oneself [27]. Self-criticism increases sensitivity to threat and reward [27]. Furthermore, sensitivity to punishment can include criticism and it relates to depression [28] and aggression [24]. Hence, sensitivity to criticism could relate to depression and aggression. Perceiving criticism in the family predicts disorganized schizotypy and positive schizotypy [29], depression and aggression [27,30,31]. Perceiving other types of social reward and punishment, such as peer rejection and peer acceptance, also relate to depression and aggression [32]. Greater aggression relates to overestimating peer acceptance, while greater depression relates to underestimating peer acceptance, but perceiving more peer rejection in adolescence [32].

Likewise, an appetite for social reward may be related to negative schizotypy, depression, and aggression [24]. Negative schizotypy and depression constitute a reduced appetite for reward [28,33–35], while aggression constitutes an excessive appetite for reward [36]. Depression and aggression have opposing propensities for experiencing social reward. Still, depressive symptoms, such as withdrawal, are associated with greater aggression when depression is associated with externalizing problems, such as bullying and having few social contacts [24]. Perceiving EE from perceiving a close relative’s emotional involvement as intrusive and perceiving less reward in standard praise could constitute a diminished appetite for social reward. Parental emotional overinvolvement relates to negative schizotypy [37]. EE-emotional overinvolvement and perceived EE-intrusiveness are similar concepts, where both concepts refer to an excessive concern for the welfare of the person showing vulnerability for mental disorder [14,38]. Perceived EE-intrusiveness relates to less sensitivity to praise and greater negative schizotypy [29]. Furthermore, perceived EE-intrusiveness relates to depression [30] and aggression as hostility [39]. Hence, perceived EE from less perceived praise, but greater perceived EE-intrusiveness, could be associated with negative schizotypy, depression, and aggression.

### Aims and hypotheses

**Figure 1.**
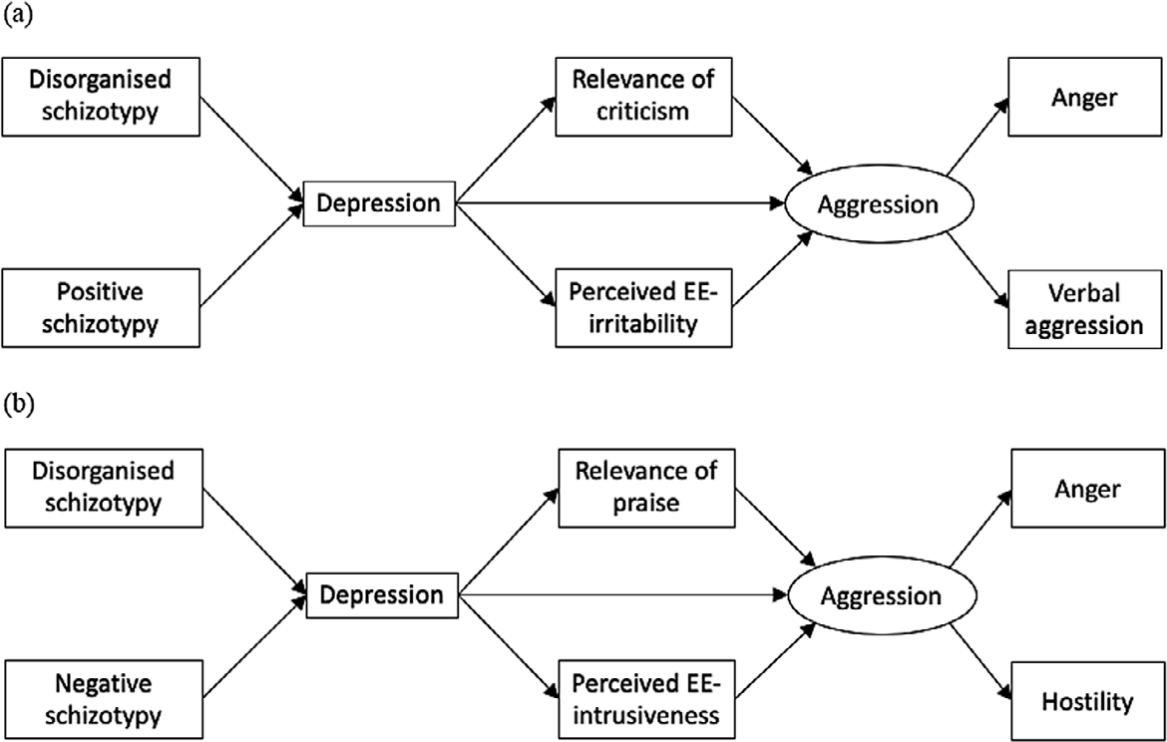
Hypothesized models of the path from schizotypy to depression and aggression, (a) from disorganized schizotypy and positive schizotypy to depression and aggression via relevance of criticism and perceived EE-irritability and (b) from disorganized schizotypy and negative schizotypy to depression and aggression via relevance of praise and perceived EE-intrusiveness. Variables in ovals are latent variables.

Two theoretical models were proposed to explain the relationship between schizotypy, depression, and aggression due to perceived EE. It was hypothesized that:

1. Disorganized schizotypy and positive schizotypy would predict greater depression and in turn severe aggression (Figure 1a). Greater perceived criticism and greater perceived EE-irritability would mediate the relationship between depression and severe aggression. Severe forms of aggression, namely anger and verbal aggression, are predicted here since the positive syndrome of schizophrenia is associated with acts of severe physical and verbal aggression more often/stronger than the negative syndrome [40,41].
2. Disorganized schizotypy and negative schizotypy would predict greater depression and in turn mild aggression (Figure 1b). Less perceived praise, but greater perceived EE-intrusiveness would mediate the relationship between depression and mild aggression. Mild aggression, namely anger and hostility, is predicted here, since the negative syndrome of schizophrenia is associated more often with milder aggression than the positive syndrome [40].

## Methods

### Participants

One hundred and four healthy participants took part. Participants were mainly young adults (80% were aged 30 years and below) and recruited by means of opportunistic sampling (75%; university students in Psychology) or social networks. The majority of this sample was characterized in an earlier study (*n* = 98) [29], with six new participants being added to the current study. Seventy-seven percent were single, 15% were cohabiting, and 8% of the sample were married. Sixty-eight percent were Caucasian, 24% were Asian, and 8% percent were African-Caribbean. Participants needed to have a close relative to participate, so that they could rate the standard criticism, the standard praise, and the Level of Expressed Emotion scale by referring to their close relative. A close relative was defined as a parent, sibling, or partner with whom the participant had face-to-face or phone contact for at least 10 h per week. Participants provided informed consent before taking part. The study was approved by the Research Ethics Committee at the University’s School of Social Sciences (No. 2013/27).

### Assessments

#### Oxford-Liverpool Inventory of Feelings and Experiences (O-LIFE)

The participants answered “Yes” or “No” to the 104 items on the O-LIFE [2,42]. The scale has four subscales, namely unusual experiences (perceptual aberrations and magical ideation), introvertive anhedonia (emotional withdrawal and lack of pleasure), Cognitive disorganization (social anxiety, moodiness, and lack of concentration), and impulsive nonconformity (lack of self-control). The subscales have good internal reliability (Cronbach’s alpha, α) in the current sample: unusual experiences, *α* = 0.90; introvertive anhedonia, *α* = 0.78; cognitive disorganization, *α* = 0.89; and impulsive nonconformity, α = 0.70.

#### Depression, Anxiety, and Stress Scale (DASS-21)

Depression is characterized by tearfulness, irritability, social withdrawal, and feeling guilty and worthless [43,44]. Participants rated the DASS by referring to their past week. Seven items concerned depression and were rated on a four-point Likert scale ranging from “Did not apply to me at all” to “Applied to me very much or most of the time.” An item on depression was “I could not seem to experience any positive feeling at all.” The depression subscale has good internal reliability in the current sample, *α* = 0.87.

#### Level of Expressed Emotion (LEE)

Participants rated their perception of their close relative over the last 3 months [38,45]. The LEE scale consists of 38 items rated on a four-point Likert scale ranging from “Untrue” to “True.” The four LEE subscales are criticism, irritability, intrusiveness, and lack of emotional support. LEE-irritability corresponds to the hostility dimension, while LEE-intrusiveness corresponds to the emotional overinvolvement dimension of the EE rating of the Camberwell Family Interview [14]. The subscales have good to excellent internal reliability in the current sample as follows, criticism_,_ *α* = 0.75; irritability, *α* = 0.79; intrusiveness, *α* = 0.81; and lack of emotional support, *α* = 0.93.

#### Buss and Perry Aggression Questionnaire (BPAQ)

Participants rated the 21 items of the BPAQ on a seven-point Likert scale, ranging from “extremely uncharacteristic of me” to “extremely characteristic of me” [46]. The questionnaire consists of four subscales, namely physical aggression, verbal aggression, anger, and hostility. Physical and Verbal aggression involve hurting or harming others. Anger denotes physiological arousal and preparing for aggression. Hostility denotes resentment, suspiciousness, and injustice. The scale has good construct validity because anger and hostility relate to emotionality [46]. Verbal aggression and anger relate to assertiveness, and verbal aggression and hostility relate to narcissism [47]. The scale has good internal reliability (Cronbach’s alpha, *α*) in the current sample, namely physical aggression, *α* = 0.88; verbal aggression, *α* = 0.85; anger, *α* = 0.86; and hostility, *α* = 0.89, total score, *α* = 0.93.

#### Affective evaluation of standard criticism and standard praise

The participants listened to 40 standard criticisms and 40 standard praises reflecting EE-criticism and positive comments from a close relative. Forty neutral comments served as a nonemotion control. Participants rated the comments for their personal relevance by answering the question, “How strongly do you relate to this comment?” on an 11-point Likert scale ranging from 0 = “not at all” to 10 = “very strongly.” Likewise, participants rated the comments for their arousal by answering the question “How arousing is this comment?” The design of this experiment has been described in full elsewhere [29]. Briefly, the style of delivering the criticism and praise verbally followed the conventions of rating a close relative for EE-criticism and EE-positive comments on the Camberwell Family Interview (the gold standard measure for rating EE) depending on tone and content of the comments [14]. Neutral comments were about the weather and scientific facts. The comments were spoken by a male and a female Psychology student who were of a similar age and level of education. The relevance of the standard criticism and the relevance of standard praise were the dependent variables in this task. Relevance captures the appraisal of emotional stimuli regardless of their valence [48] and reflects perceived family EE better than arousal [29]. The median of the ratings of the relevance of the 40 comments in each condition (criticism, praise, and neutral comments) was calculated.

### Statistical analysis

**Figure 2.**
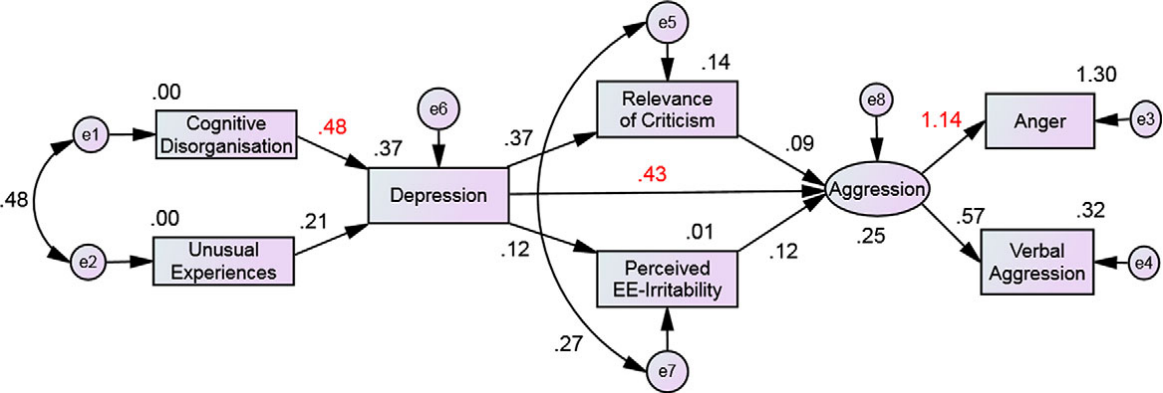
Modified path model of the path from disorganized schizotypy and positive schizotypy to depression and aggression via perceived criticism and perceived EE-irritability. Notes: Values represent standardized coefficients; values in red represent the highest standardized path coefficients at each stage of the path; values besides observed variables (in boxes) are squared multiple correlations; values besides straight arrows are indirect, direct and total effects; values next to curved arrows are error covariances between endogenous variables; variables in circles or ovals are unobserved variables (latent variables or error variances).

**Figure 3.**
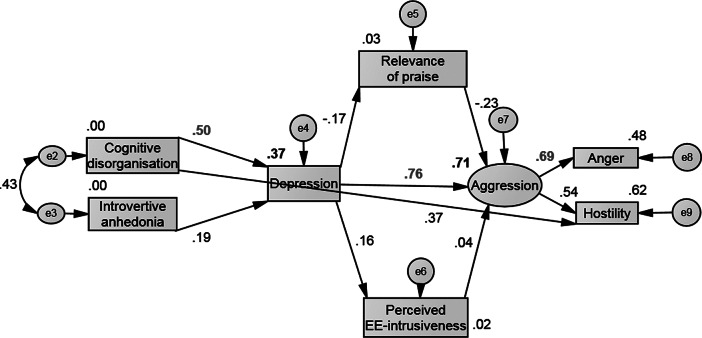
Modified path model of the path from disorganized schizotypy and negative schizotypy to depression and aggression via perceived praise and perceived EE-intrusiveness. Notes: Values represent standardized coefficients; values in red represent the highest standardized path coefficients at each stage of the path; values besides observed variables (in boxes) are squared multiple correlations; values besides straight arrows are indirect, direct and total effects; values next to curved arrows are error covariances between endogenous variables; variables in circles or ovals are unobserved variables (latent variables or error variances).

Skewness and kurtosis were in the normal range for all variables, ranging from −0.48 to 1.18 for skewness and from −0.93 to 1.15 for kurtosis. For exploratory purposes, two-tailed Pearson correlations were performed in SPSS, version 24, between the schizotypy subscales, median relevance of criticism, median relevance of praise, perceived EE, depression, and aggression. Path analyses were performed in SPSS Amos, version 25. Two models specified the path from schizotypy to aggression via depression and perceived EE. The first model tested the first hypothesis of the path from cognitive disorganization and unusual experiences to depression. The path continued from depression to aggression via relevance of criticism and LEE-irritability, where aggression was the latent variable and anger and verbal aggression were the observed variables (Figure 2). The second model tested the second hypothesis of the path from cognitive disorganization and introvertive anhedonia to depression. The path continued from depression to aggression via relevance of praise and LEE-intrusiveness, where aggression was the latent variable and anger and hostility were the observed variables (hypothesis 2, Figure 3). Maximum likelihood estimation and, due to the small sample size, a boot strapping procedure of 2,000 samples were used to calculate the parameters of the path model, including standardized indirect effects and their 90% confidence intervals. Several fit indices determined the criterion for a good fit between the hypothesized and observed models [49]. A nonsignificant chi-square test indicates good fit. A good comparative fit index has a value >0.95 and indicates that the hypothesized model fits the data well. A root-mean square error of approximation value of <0.05 indicates good fit, with values between 0.08 and 0.1 representing mediocre fit and a value >0.1 indicating poor fit. The standardized root mean square residual represents the average value across all standardized residuals, and a value of <0.05 indicates good fit. The Akaike Information Criterion requires the fit statistic of the respecified model to be lower than that of the hypothesized (default) model. The model was respecified by studying the modification indices and standardized residuals and introducing covariances between unobserved variables (error variance) and/or introducing a direct path between the observed variables [50].

## Results

### Sample characteristics

Participants were mainly young (mean age 23.84 ± 5.86 years) and female (79%) and single (77%). Compared to the normative sample of females (*n* = 237) aged 18 to 21 years [42], the current sample had higher introvertive anhedonia (*t*(339) = 4.4, *p* < 0.001), but did not differ on unusual experiences (*t*(339) = 1.69, *p* = 0.09), cognitive disorganization (*t*(339) = 0.77, *p* = 0.44) and impulsive nonconformity (*t*(339) = 0.04, *p* = 0.96).

### Correlations between schizotypy, aggression, perceived EE, and depression

O-LIFE unusual experiences (positive schizotypy) correlated positively with the relevance of criticism, LEE-criticism and LEE-irritability, depression, physical aggression, verbal aggression, anger and hostility (Table 1). O-LIFE introvertive anhedonia (negative schizotypy) correlated positively with relevance of criticism, LEE-criticism, LEE-irritability and LEE-intrusiveness, depression, anger and hostility, and negatively with relevance of praise. Cognitive disorganization (disorganized schizotypy) correlated positively with relevance of criticism, LEE-criticism, LEE-irritability, LEE-intrusiveness, depression, anger and hostility, and negatively with relevance of praise.

**Table 1.** Descriptive statistics and Pearson correlations (paths *a* and *c*′, direct effect, of the mediation analyses) between schizotypy, aggression, perceived criticism and praise, depression, and perceived expressed emotion.

	Mean (SD)	1	2	3	4
1. O-LIFE—UE	8.93 (6.47)	–			
2. O-LIFE—IA	7.18 (4.45)	0.077	–		
3. O-LIFE—CD	13.21 (6.17)	0.48***	0.43***	–	
4. O-LIFE—IN	9.25 (3.77)	0.46***	0.12	0.46***	–
5. Relevance of criticism	4.83 (2.50)	0.26**	0.23*	0.41***	0.35***
6. Relevance of praise	5.83 (2.03)	0.05	−0.21*	−0.24*	−0.16
7. LEE-criticism	9.28 (3.08)	0.25**	0.2*	0.32**	0.37***
8. LEE-irritability	11.64 (3.67)	0.22*	0.23*	0.27**	0.27***
9. LEE-intrusiveness	18.7 (5.23)	0.15	0.26**	0.22*	0.18
10. LEE-LES	31.86 (10.02)	0.13	0.17	0.1	0.36**
11. DASS-depression	9.94 (9.15)	0.44***	0.41***	0.58***	0.41***
12. BP-physical aggression	23.51 (11.40)	0.20*	0.15	0.12	0.51***
13. BP-verbal aggression	17.48 (7.31)	0.20*	−0.02	0.11	0.39***
14. BP-anger	19.74 (9.25)	0.29**	0.27**	0.31**	0.52***
15. BP-hostility	24.33 (11.86)	0.43***	0.36***	0.64***	0.50***

Abbreviations: BP, Buss and Perry aggression questionnaire; DASS, depression, anxiety and stress scale; LEE, level of expressed emotion scale; LEE-LES, lack of emotional support; O-LIFE, Oxford-Liverpool Inventory of Feelings and Experiences; O-LIFE–CD, O-LIFE cognitive disorganization; O-LIFE–IA, O-LIFE introvertive anhedonia; O-LIFE–IN, O-LIFE impulsive nonconformity; O-LIFE–UE, O-LIFE unusual experiences.

* *p* < 0.05.

** *p* < 0.01.

*** *p* < 0.001.

### Path from schizotypy to depression and aggression via relevance of criticism and perceived EE-irritability

The initial model indicated that the hypothesized model did not fit the data well (Table 2). To achieve acceptable goodness-of-fit, error covariances were specified based on the modification indices between (a) cognitive disorganization and unusual experiences subscales of the O-LIFE, (b) relevance of criticism and LEE-irritability, and (c) anger and verbal aggression subscales of the BPAQ (Figure 2). The error variances of the O-LIFE subscales are expected to covary because they share similar psychometric properties. For the same reason, the error variances of subscales of the BPAQ are expected to covary. Hence, error covariances were applied to these pairs of observed variables. The covariance between the error variance of relevance of criticism and that of LEE-irritability is plausible because relevance of criticism and LEE-irritability are both measures of perceived EE-hostility [29].

**Table 2.** Fit indices for the hypothesized and respecified models of the path from schizotypy to depression and aggression.

	CD and UE to aggression via depression, Relevance of criticism, and LEE-irritability		CD and IA to aggression via depression, perceived praise, and perceived EE-intrusiveness	
	Hypothesized model	Respecified model	Hypothesized model	Respecified model
Chi-square	49.44	15.23	54.24	15.21
Df	12	10	12	10
*P*	<0.001	0.12	<0.001	0.125
CFI	0.81	0.97	0.78	0.97
GFI	0.88	0.96	0.87	0.96
RMSEA	0.17	0.07	0.18	0.07
90% lower CIs	0.12	<0.001	0.14	<0.001
90% upper CIs	0.23	0.14	0.24	0.14
SRMR	0.14	0.67	0.14	0.07
AIC				
Default model	81.44	51.23	86.24	51.21

Abbreviations: AIC, Akaike Information Criterion; CD–O-LIFE, Oxford-Liverpool Inventory of Feelings and Experiences cognitive disorganization; CFI, comparative fit index; CIs, confidence intervals; Df, degrees of freedom; GFI, goodness of fit index; RMSEA, root mean square error of approximation; IA, O-LIFE introvertive anhedonia; SRMR, standardized root mean square residual; UE, O-LIFE unusual experiences.

The following direct paths were significant and are mentioned in decreasing order of the size of the standardized path coefficients, namely anger to aggression (*r* = 1.14, *p* < 0.001; note that a standardized regression coefficient >1 is legitimate in a path analysis [51]), aggression to verbal aggression (*r* = 0.57, *p* < 0.001), cognitive disorganization to depression (*r* = 0.48, *p* < 0.001), depression to aggression (*r* = 0.42, *p* < 0.001), Depression to relevance of criticism (*r* = 0.37, *p* < 0.001), and unusual experiences to depression (*r* = 0.21, *p* = 0.017). The following indirect paths were significant and are mentioned in decreasing order of the size of the standardized path coefficients, namely depression to anger (*r* = 0.54, *p* = 0.001), depression to verbal aggression (*r* = 0.27, *p* = 0.001), cognitive disorganization to anger (*r* = 0.26, *p* = 0.001), cognitive disorganization to aggression (*r* = 0.23, *p* < 0.001), cognitive disorganization to relevance of criticism (*r* = 0.18, *p* = 0.001), cognitive disorganization to verbal aggression (*r* = 0.13, *p* < 0.001), and unusual experiences to verbal aggression (*r* = 0.06, *p* = 0.47).

### Path from schizotypy to depression and aggression via relevance of praise and perceived EE-intrusiveness

The initial model indicated that the hypothesized model did not fit the data well (Table 2). An error covariance was specified between cognitive disorganization and introvertive anhedonia as the modification indices indicated that this modification would improve the fit of the model. These observed variables are expected to be related because they are subscales of O-LIFE (Figure 3). A direct path was specified from cognitive disorganization to BPAQ-hostility. This modification is meaningful, since cognitive disorganization predicts aggression [26]. The respecified model achieved acceptable goodness-of-fit. The following direct paths were significant, namely depression to aggression (*r =* 0.76, *p* < 0.001), aggression to anger (*r* = 0.69, *p* < 0.001), aggression to hostility (*r* = 0.54, *p* < 0.001), cognitive disorganization to depression (*r =* 0.5, *p* < 0.001), cognitive disorganization to hostility (*r =* 0.37, *p* < 0.001), relevance of praise to aggression (*r* = −0.23, *p* = 0.019), and introvertive anhedonia to depression (*r* = 0.0.19, *p* = 0.024). The following indirect paths were significant, namely from depression to anger (*r* = 0.56, *p* = 0.001), depression to hostility (*r* = 0.43, *p* = 0.002), cognitive disorganization to aggression (*r* = 0.4, *p* = 0.001), cognitive disorganization to anger (*r* = 0.28, *p* = 0.001), cognitive disorganization to hostility (*r* = 0.22, *p* = 0.001), introvertive anhedonia to aggression (*r* = 0.16, *p* = 0.008), relevance of praise to anger (*r* = −0.16, *p* = 0.011), introvertive anhedonia to anger (*r* = 0.11, *p* = 0.008), introvertive anhedonia to hostility (*r* = 0.08, *p* = 0.006), and introvertive anhedonia to relevance of praise (*r* = −0.03, *p* = 0.04).

## Discussion

People with a significant level of schizotypy, depression, and aggression are reported to perceive high EE from their close relative [29,30]. Furthermore, disorganized, positive and negative dimensions of schizotypy and/or schizophrenia predict aggression [11,24,41]. This is the first study to collectively examine the role of perceived EE in relationship with schizotypy, depression, and aggression. Two path models predicted the path from schizotypy to depression and aggression, one via perceived EE as perceived criticism and LEE-irritability and the other via perceived EE as perceived praise and LEE-intrusiveness. In both models, disorganized schizotypy (O-LIFE cognitive disorganization) was the strongest of the schizotypal traits to predict the path from schizotypy to aggression.

### The path from disorganized schizotypy to depression and aggression

Social anxiety could mediate the relationship between disorganized schizotypy and aggression. Disorganized schizotypy denotes social anxiety and communication disorder [52]. Disorganized schizotypy also relates to social stress [53–55] and social anxiety as rejection sensitivity [56]. In turn, disorganized schizotypy and social anxiety relate to poor recognition of anger [57,58]. Hence, aggression in schizotypy could be reactive to peer victimization [11]. Disorganized schizotypy directly and indirectly predicted aggression as hostility, anger, and verbal aggression in the current study. Hence, social anxiety, such as sensitivity to criticism and rejection, may mediate the relationship between disorganized schizotypy and aggression. Disorganized schizotypy relates to accepting unfair social rewards [59] and aggression [60]. In turn, rejection sensitivity increases the likelihood of retaliation [61]. Given that disorganized schizotypy also features lack of concentration and attentional deficits [62], it may be interesting to explore disorganized schizotypy in relation to attention deficit hyperactivity disorder which has been linked to antisocial behavior through inattention and impulsivity [63].

### The path from positive schizotypy and negative schizotypy to depression and aggression

Positive schizotypy (O-LIFE unusual experiences) directly predicted depression and indirectly predicted verbal aggression in the current study. Disorganized schizotypy and depression predicted the relevance of criticism. Having a high level of depression due to excessive negative metacognitive beliefs, such as being self-critical and perceiving criticism in others, could mediate the relationship between positive schizotypy and aggression. Consistent with this notion, perceived victimization from peers has been found to mediate the relation between positive schizotypy and aggression [11]. Relevance of criticism could be seen as sensitivity to punishment. The behavioral inhibition system (BIS) and behavioral approach system (BAS) are two theoretical systems that could account for sensitivity to punishment and reward in criticism and praise, respectively, and explain the relationship between schizotypy and aggression [64,65]. The heighted BIS activation may account for sensitivity to punishment [33], such as self-criticism [27], and may explain the role of perceived criticism in mediating the relationships between positive schizotypy, depression, and aggression. Likewise, negative schizotypy (O-LIFE introvertive anhedonia) directly predicted depression and indirectly predicted the relevance of praise and aggression as hostility and anger in the current study. The BAS accounts for sensitivity to reward and the tendency to approach situations [33]. The BAS puts psychoticism (a personality dimension that includes schizotypal traits) on a continuum with psychopathy (a personality dimension concerned with aggression) [64]. Diminished BAS from praise in negative schizotypy may predict depression and aggression because BAS mediates the link between psychoticism and psychopathy [64]. Future research could test the role of BIS/BAS activation in the relationships between perceived EE, schizotypy, depression, and aggression.

### Limitations, future directions and implications

The findings may be considered exploratory given the sample size (*n* = 104). Although bootstrapping tests meant that the models were tested in 2,000 hypothetical samples, further research could test the respecified models in a larger sample and also explore possible gender differences in the path from schizotypy to depression and aggression, given that depression relates to being a victim of violence in women but being the perpetrator in men [66]. Our participants were mostly university students studying psychology who may be better acquainted with the psychological concepts being investigated than the nonacademic population. Therefore, the findings may not generalize to the wider subclinical population. Lastly, future research may test–retest reliability of the affective evaluation task.

Our findings have implications for schizophrenia. Thought disorder in schizophrenia is the clinical analogue of disorganized schizotypy [67]. Thought disorder comprises peculiar language, illogical thinking, and loose association [52,68]. Patients with schizophrenia who have had a past criminal conviction and current thought disorder are more likely to be disorganized and act violently than patients who do not have thought disorder [26]. These previous findings, taken together with our findings, i.e., disorganized schizotypy predicting aggression through perceived criticism and less perceived praise, suggest that aggression in patients with thought disorder may be reactive to perceived family criticism/lack of praise and associated anger and stress. Thus, thought disorder and the reactive nature of aggression should be considered when supporting patients with schizophrenia with a history of aggression and violence. Our findings also appear to be consistent with previous data showing that intensification of anger explains the link between positive symptoms and violence [41].

In conclusion, disorganized schizotypy, and to a lesser extent positive schizotypy and negative schizotypy, predicts depression and aggression. Greater perceived criticism, but less perceived praise, in family communication seems to explain the path from schizotypy to depression and aggression. Given that disorganized schizotypy is analogous with thought disorder in schizophrenia, schizophrenia patients with thought disorder tend to be disorganized and act violently than those without thought disorder [26]. There is a need to consider thought disorder-EE link as a potential contributor to depression and aggression in people with schizophrenia.
